# Growth characterization of CHO DP-12 cell lines with different high passage histories

**DOI:** 10.1186/1753-6561-5-S8-P29

**Published:** 2011-11-22

**Authors:** Christoph Heinrich, Timo Wolf, Christina Kropp, Stefan Northoff, Thomas Noll

**Affiliations:** 1 Institute of Cell Culture Technology, Bielefeld University, Bielefeld, Germany; 2 TeutoCell AG, Bielefeld, Germany

## Introduction

For industrial pharmaceutical protein production fast growing, high producing and robust cell lines are required. To select pH-shift permissive and faster growing sub-populations, the CHO DP-12 cell line was serially subcultured for more than four hundred days in shaker flasks. Initial adaptation to growth in suspension was carried out in chemically defined medium without hypoxanthine and thymidine (HT), while the final medium used for long term cultivation contains HT. Cell samples were cryopreserved at four different time points after 21, 95, 165 and 420 days. Cultivations of these four sub-populations (SP) in shaker flasks and bioreactors revealed considerable differences in specific growth rates and product formation as well as in the metabolism of glucose, lactate and several amino acids. For the elucidation of the intracelluar mechanism behind these alteration in growth characteristics and metabolism additional probes were analyzed using proteomic and metabolomic approaches [1].

## Material and methods

In this study the CHO DP-12 clone#1934 (ATCC CRL-12445) was used as reference organism. It co-expresses the variable light and heavy chains of the murine 6G4.2.5 monoclonal antibody (ATCC-HB-11722) which inhibits binding of interleukin 8 to human neutrophile. CHO DP-12 cells were cultivated in CD-ACF medium TC 42 (TeutoCell AG) and PowerCHO-2 (LONZA AG) for the first steps of suspension adaptation. 200 nM methotrexate was present at any time. Precultures and parallel cultivations were carried out in 125 mL and 250 mL polycarbonate Erlenmeyer flasks (Corning Life Sciences). Incubator conditions were set to 37°C, 5% CO_2_ and relative humidity of 80%. A shaker revolution of 185 rpm or 125 rpm with an orbital movement of 2" was chosen. For bioreactor cultivations four parallel vessels (Applikon) controlled by CellfermPro 2.3 software (DASGIP AG) were used. Cultivation parameters were set to 37°C, 40% DO and pH 7.1.

Cell concentration and viability were determined with a CEDEX system (Innovatis-Roche AG). The anti IL-8 antibody was quantified using Protein A HPLC.

A MACSQuant^®^ Analyzer (Miltenyi Biotec GmbH) was used for the measurements of intracellular IgG-product pools. Intracellular detection of the antibodies required permeabilization of the cell membrane with detergents. IgG light and heavy chains were stained with fluorochrome-conjugated antibodies that bind to Fc and kappa chains of IgGs within fixed CHO cells (all solutions from Miltenyi Biotec GmbH).

## Results

The initial adaptation to growth in suspension of the CHO DP-12 cells led to considerable changes in average cell diameter and specific growth rate. Within the first 100 days of serial subculturing the cell diameter dropped from a maximum of about 17 µm to 12 µm as the lowest value. In the same period the cell specific growth rate increased from initially 0.2 d^-1^ up to values greater than 1.0 d^-1^. After further 320 days cells had an average diameter about 14 ± 0.74 µm and a mean specific growth rate of 0.82 ± 0.12 d^-1^.

**Figure 1:**
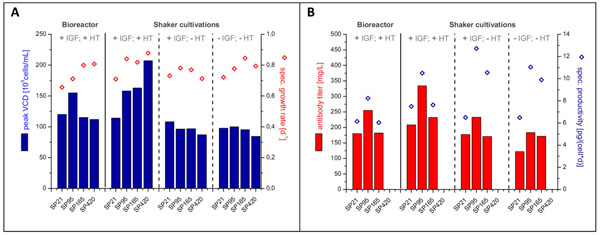
Overview of growth performance (A) and product formation (B) of the four sup-populations in controlled and uncontrolled culture systems with varying media supplementation.

The growth characterizations of the four sub-populations SP21, SP95, SP165 and SP420 were carried out in several parallel controlled and uncontrolled batch cultivations. In addition to the influence of the insulin like growth factor 1 (IGF) the presence of hypoxanthine and thymidine (HT) was investigated as well. Most important characteristics for growth and productivity determined in this experimental setup are presented in Figure 1.

Though the sub-populations SP95, SP165 and SP420 seemed to possess comparable growth rates in shake flask cultivations, they differed remarkably in their maximum cell density when HT-mix was present. For SP21 and SP420, maximum cell densities of about 1.1·10^7^ cells/mL and 2.1·10^7^ cells/mL, respectively, were determined. These results indicate an influence of the HT-mix on the maximum reachable cell densities. Furthermore, an increased passage number seemed to cause decreasing growth performance in the controlled bioreactor system compared to the results from the shake flask cultivations with HT containing medium. This fact could be associated to the adaptation of subcultivated cells to pH-shifts that occur during cultivation under uncontrolled conditions. Additionally, the observed differences between the four sub-populations in terms of the metabolism of glucose, lactate and several amino acids might play a role in this context. In bioreactor cultivations a two-fold higher lactate formation was observed for SP420 as compared to SP21, for instance.

The highest specific production rate of 10.5 pg/(cell·day) was obtained for the sub-population SP95 resulting in a final antibody titer of 334 mg/L. Considering long-term cultivation, cell specific productivity increased during first passages and was lost with ongoing subcultivation. Further cytometry analysis regarding intracellular productivity of examined CHO DP-12 cells revealed that serial subculturing resulted in accumulation of a subclone expressing only the light chain of the IL-8 antibody.

## Conclusions

Serial subculturing over an extended period led to the selection of faster growing and pH-shift permissive cells, thus resulting in higher viable cell densities, especially in uncontrolled shake flask cultivations. Furthermore, cells or rather sub-populations with distinct metabolic characteristics were enriched along the subculturing process. This indicated that a targeted experimental approach could be used e.g. to specifically select cells adapted to low glutamine concentrations and therefore, a reduced consumption rate. On the other hand, the observed loss of productivity shows that the selection pressure given by 200 nM methotrexate and deprivation of hypoxanthine and thymidine could not prevent an increase of sub-populations expressing no or only incomplete (non-native, deficient) product. This problem could be caused by the vector design maybe used for the CHO DP-12 cells, which resulted in a non associated integration of the dihydrofolate reductase (dhfr) and anti-IL 8 sequences. Hence, it might be interesting to monitor the intracellular expression during clone selection or even seed-train development by flow cytometry.
